# Factors Associated with the Clinical Course of Vitreomacular Traction

**DOI:** 10.1155/2020/9457670

**Published:** 2020-12-21

**Authors:** Petros Petrou, Evangelia Chalkiadaki, Marie-Helene Errera, Sidath Liyanage, Louisa Wickham, Evangelia Papakonstantinou, Aristotelis Karamaounas, Menelaos Kanakis, Ilias Georgalas, Stylianos Kandarakis, David Charteris

**Affiliations:** ^1^First Ophthalmology Department, “G. Gennimatas” Hospital, National and Kapodistrian University of Athens, Athens, Greece; ^2^University of Pittsburg, Pittsburgh, PA, USA; ^3^Bristol Eye Hospital, Bristol, UK; ^4^Moorfields Eye Hospital, London, UK

## Abstract

**Background:**

To analyze the optical coherence tomography (OCT) characteristics as well as the clinical and demographic features to investigate their possible role to the course of vitreomacular traction syndrome.

**Methods:**

The inclusion criteria were vitreomacular adhesion with traction causing distortion of the retinal architecture, with or without the presence of an epiretinal membrane, regardless of the size of the adhesion; age >18 years; follow-up of at least three months; and adequate quality OCT scan. Measurements of foveal thickness, average macular thickness, macular volume, maximum vertical and horizontal vitreomacular adhesion, nasal and temporal angles of traction, hyaloid hyperreflectivity, the presence of an epiretinal membrane (ERM), and cone outer segment tips detachment were obtained.

**Results:**

150 eyes were included in the analysis. 36 eyes (24%) developed complete resolution at the last visit, 19 eyes (12.7%) formed a full-thickness macular hole, and 95 eyes (63.3%) showed no resolution of the traction. Better BCVA at the first visit was associated with an increased likelihood of resolution of the VMT, but increasing age, CMT, and BCVA in the end of the follow-up was associated with a reduction in the likelihood of resolving. Of the other variables that were studied, no statistical significant predictors were identified.

**Conclusions:**

Better BCVA in the first visit was associated with an increased likelihood of resolution of the VMT that occurred in 24% of our cases. Other factors such as the vertical area of adhesion and the angle of adhesion were not identified as prognostic factors affecting the clinical course of the disease.

## 1. Ιntroduction

Abnormalities of the vitreoretinal interface are involved in the pathogenesis of several macular conditions. At birth, most eyes have complete vitreoretinal attachment throughout the fundus and vitreous gel remains fully attached to the internal limiting membrane until young adulthood. After the age of 40, there is a significant decrease in the gel volume and a concurrent increase in the liquid volume in the central vitreous primarily. The significant alterations in vitreous structure that occur with aging and with certain diseases [1] can result in posterior vitreous detachment, which can either be normal/complete or anomalous/incomplete. Anomalous posterior vitreous detachment results from gel liquefaction without concurrent weakening of vitreoretinal adherence, causing clinical manifestations based upon where vitreous is most liquefied and where the interface is most firmly adherent.

The persistent attachment of vitreous at the macular region may cause anteroposterior macular traction that can lead to or exacerbate at least seven distinct macular pathologies, including idiopathic epiretinal membrane (ERM), macular microhole, idiopathic macular hole (MH), tractional cystoid macular edema (CME), tractional diabetic macular edema (DME), vitreomacular traction syndrome (VMTS), and myopic macular retinoschisis. The complication that occurs in an eye with perifoveal vitreous detachment is likely determined in part by the size and strength of the residual vitreomacular adhesion and perhaps by the structural integrity of the foveal stroma [2]. If the foveal morphologic features remain unperturbed, the term vitreomacular adhesion (VMA) is used. Vitreomacular traction (VMT), on the other hand, is characterized by anatomic distortion of the fovea, which may include pseudocysts, macular schisis, cystoid macular edema, and subretinal fluid [3]. VMT can be relatively asymptomatic or present with decreased vision, metamorphopsia, photopsia, and central scotoma [2, 4]. Although VMA is generally considered a part of the natural course of PVD, a recent observational cohort study showed that VMA released spontaneously in only 30% of patients, while 53% remained stable and 16% progressed from VMA to develop VMT or full-thickness macular hole [5].

Until the advent of optical coherence tomography (OCT), it was difficult to visualize the vitreofoveal interface routinely, and various indications of the role of the vitreous had been given in the literature. During the past two and a half decades, OCT has evolved to become an essential tool in diagnosing, understanding, and monitoring the progression of vitreomacular interface pathologies. OCT allows detailed assessment of the retinal microstructure associated with vitreomacular adhesion or traction, which visualizes their effect on the retina and allows for a more accurate visual prognosis [6]. Vitreomacular traction can be subclassified by the diameter of vitreous attachment to the macular surface as measured by OCT, with attachment of 1500 *μ*m or less defined as focal and attachment of more than 1500 *μ*m as broad. When associated with other macular diseases, VMT is classified as concurrent [3].

Serial observations have confirmed that the vitreomacular traction will resolve spontaneously in 11–53% of cases [4, 7–10]. It is not known, however, what factors might be associated with this spontaneous resolution or the progression to MH/VMT causing vision loss. Our study was designed to analyze detailed OCT characteristics as well as clinical and demographic features of our population to investigate the possible role of them to the clinical course of the disease.

## 2. Methods

We conducted a retrospective observational study of the clinical course of vitreomacular traction syndrome of 150 eyes at Moorfields Eye Hospital, London, UK, between January 2008 and November 2013. The study had institutional ethical committee approval and conformed to the guidelines of the Declaration of Helsinki.

### 2.1. Identification of Cases

The clinical records of all patients who were recorded in our electronic patient record system and were diagnosed with VMTS, as well as patients in whom the terms “VMT” or “vitreomacular” appeared in their electronic letters within the specified date range were reviewed. Inclusion criteria included vitreomacular traction causing distortion of the retinal architecture, with or without the presence of an epiretinal membrane, regardless of the size of the adhesion; age >18 years; follow-up of at least three months; and adequate quality OCT scan. The exclusion criteria included the presence of a full- or partial-thickness macular hole and coexistent ocular diseases affecting the macula (i.e., diabetic maculopathy, retinal vein occlusion, age-related macular degeneration, and uveitis). Moreover, patients who eventually underwent vitrectomy for vitreomacular traction (not macular hole) were excluded from the analysis.

### 2.2. OCT Scans and Measurements

All patients had OCT scans using the 6 × 6 mm cube protocol (512 × 128 scan) on the Topcon 3D OCT 1000 or 2000 series (Topcon Corporation, Tokyo, Japan). Measurements of foveal thickness, average macular thickness, and macular volume were obtained using the in-built software of the OCT devices. In scans where the ILM was incorrectly traced by the software, this was manually corrected using the in-built software. In cases where manual correction was not possible, the foveal thickness was measured with the caliper provided. In these cases, the average thickness and macular volume data were deemed inaccurate and were excluded from the analysis. The maximum horizontal vitreomacular adhesion was measured with the caliper, and the maximum vertical adhesion was calculated by measuring the number of cuts where adhesion was present and multiplying this by 0.046875 (6 mm/128 OCT cuts).

**Figure 1:**
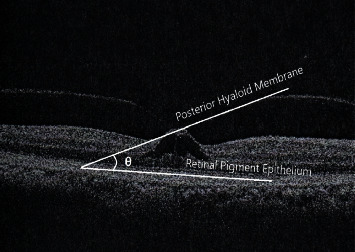
Time-domain optical coherence tomography of a patient with vitreomacular traction. The nasal angle is measured in this case by using the NIH image tool ImageJ. The white lines define the measurement of the vitreofoveal angle (*θ*). A line parallel to the RPE and another line tangential to the posterior hyaloid at the point of adhesion are traced. The angle between the two lines is measured.

The nasal and temporal angles of traction were measured by using the NIH image tool ImageJ at a horizontal scan of OCT passing through the fovea. A line parallel to the RPE was traced on both the nasal and temporal points of adhesion of the posterior hyaloid to the retina. Another line tangential to the posterior hyaloid at the point of adhesion was traced. The angle between the two lines was measured using the angle tool (Figure 1).

Hyaloid hyperreflectivity, the presence of an epiretinal membrane (ERM), and cone outer segment tips detachment (COST) were also noted. The COST line was observed as a highly reflective line between the IS/OS line and retinal pigment epithelium. All measurements were obtained by the same observer.

### 2.3. Other Data

All patients underwent a thorough ophthalmologic examination, including best-corrected visual acuity (BCVA) measurements by means of Snellen charts, slit-lamp biomicroscopy, dilated fundoscopy, and OCT. Data were collected and analyzed regarding the demographic characteristics, the length of follow-up, the coexistence of diabetes, and the presence of macular hole in the other eye. The BCVA was converted to the logarithm of the minimum angle of resolution (logMAR) for statistical purposes.

### 2.4. Statistical Analysis

The statistical analysis was performed using SPSS 22.0 statistical software (SPSS Inc., Chicago, IL, USA). Descriptive statistics, including the mean values, median, standard deviations, and percentages, were used to analyze the baseline characteristics in all patients. Variables with more than 5% of missing data were excluded. A multiple linear regression analysis (stepwise backward elimination of nonsignificant variables (*P* ≥ 0.05)) was used to examine the effect of parameters on BCVA in the beginning of follow-up and on BCVA at the end of the follow-up. A binary logistic regression model was used for prognostic prediction of VMT release.

## 3. Results

VMT was identified in 150 eyes. Fifty-seven (38%) of the patients were men, and 93 (62%) of the patients were women. The mean length of follow-up was 20.09 ± 13.44 months (mean ± SD) (range 3–54 months). Mean age was 71.9 ± 10.5 years (mean ± SD) (range 49–95) at initial examination. The mean logarithm of the minimum angle of resolution (logMAR) BCVA at the initial visit was 0.26 ± 0.22 (mean ± SD) and improved to 0.28 ± 0.28 at the last visit. The mean age of patients presenting with this condition was 71.93 years, with women comprising the majority of cases (62%). The mean baseline visual acuity was 0.26 logMAR units, and the central macular thickness was 308.82 *μ*m. The detailed characteristics of the analyzed features and baseline characteristics are presented in Table 1.

Of the 150 eyes with vitreomacular traction at the initial visit, based on the anatomical results of the last OCT examination, we categorized

- 36 eyes (24%) that developed complete resolution of the traction without macular hole formation at the last visit (R-VMT)
- 19 eyes (12.7%) that formed full-thickness macular hole (FTMH) stage ΙΙ or ΙΙΙ and
- 95 eyes (63.3%) that showed no resolution of the traction (NR-VMT) by the end of our follow-up period

Multiple variable linear regression analysis demonstrated that the BCVA in the beginning of our follow-up was the only parameter affecting the final visual outcome (post-BCVA = 0.115 + 0.694 × pre-BCVA). It also showed that BCVA at the beginning of our follow-up was positively affected by age and central macular thickness, but negatively affected by the presence of COST detachment and the average thickness in the macula.

**Figure 2:**
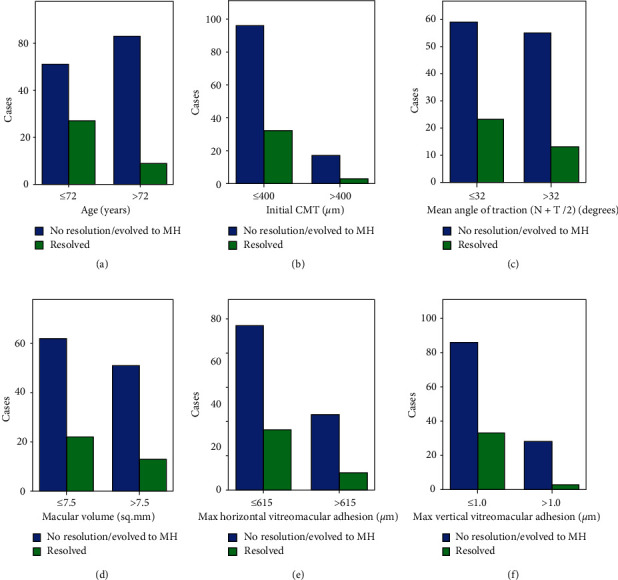
Graphs showing the number of cases that developed complete resolution of the VMT or either evolved to macular hole stage II or III or showed no resolution of the traction in relation to various baseline factors (*A,* age; *B,* initial central macular thickness; *C,* mean (nasal and temporal) angle of resolution; *D,* macular volume; *E,* maximum horizontal vitreomacular adhesion; and *F,* maximum vertical vitreomacular adhesion).

**Figure 3:**
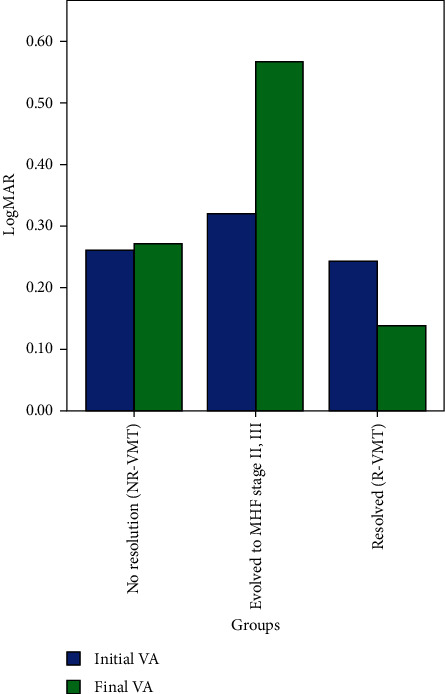
Graph showing the initial and final visual acuities for groups in which vitreomacular traction syndrome remained stable (NR-VMT), progressed to full-thickness macular hole stage II or III (MHF), and developed complete resolution of the traction without macular hole formation at the last visit (R-VMT).

**Figure 4:**
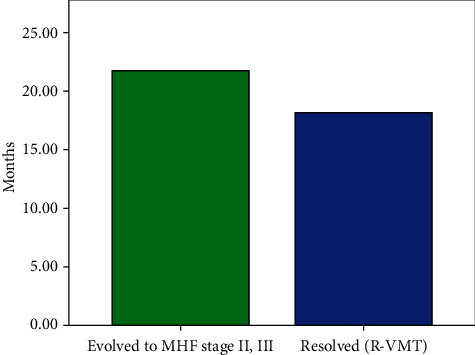
Time taken for cases to evolve to full-thickness macular hole stage II or III (MHF) or develop complete resolution of the traction without macular hole formation (R-VMT).

We further categorized our study group in 36 eyes that developed complete resolution of the VMT (defined as a release of the vitreomacular adhesion from all macular points seen on all OCT scans) and 114 eyes that either evolved to FTMH stage II or III or showed no resolution of the traction. The mean time of resolution of VMT was 16 ± 14 months (range 2–50 months). Also, the mean time of macular hole formation was 17 ± 15 months We used these two generalized groups so as to create a binary logistic regression model for prognostic prediction of VMT release. Of the 150 eyes that were identified, 135 eyes were included in the analysis due to the missing data of the rest of the cases. This binary logistic regression model showed that better BCVA in the first visit was associated with an increased likelihood of resolution of the VMT, but increasing age, CMT, and BCVA in the end of the follow-up was associated with a reduction in the likelihood of resolving. Therefore, increasing age, thicker macula, and better final BCVA were associated either with the stability of the condition of the traction or with progression to macular hole (Figures 2–4).

The binary logistic regression model was also performed to ascertain the effects of our study variables on the likelihood of not resolving the traction or evolving to FTMH. We found that there were no statistical significant predictors in this case.

## 4. Discussion

Although VMT syndrome has been recognized by ophthalmologists for decades, we are now able to examine, observe, and understand the natural course of VMT with the aid of OCT imaging. Studying the processes occurring during several months of observation of VMT cases, it is crucial to assess factors influencing the decision of observation, pharmacologic vitreolysis, or eventual vitrectomy as a method of treatment for this disease. Although many authors have analyzed the natural course of vitreomacular traction syndrome, the effort to identify the potential prognostic morphological and clinical factors has not been significantly fruitful to date. In our study, we observed 150 eyes with VMT syndrome by OCT for a mean follow-up period of 20 months. We found 24% eyes with a complete resolution of the traction, 12.7% eyes that formed FTMH, and 63% that showed no resolution of the traction by the end of the follow-up period. When comparing our results to the previously published series, we observed, in accordance with our findings, that most of VMT syndromes persist over time, approximately 60% of them, 5–17% develop FTMH, and 17–32% of the eyes develop a full resolution [4, 8, 11–17].

Hikichi et al. [8] reported complete PVD in 6 of 53 eyes (11%) with VMTS without using OCT, during an average follow-up period of 15 months. Larsson [11] found persistent VMTS by OCT among 11 eyes during the 12-week period before vitrectomy, with the advent of SD-OCT. Odrobina et al. [12] observed spontaneous resolution of VMT in the eyes with less vitreous surface adhesion and without ERMs. They evaluated 19 eyes with VMTS for an average of 8 months and reported complete PVD in 47% of the eyes, FTMH formation in 5% of the eyes, and lamellar macular hole formation in 5% of the eyes. Zhang et al. [10] studied 23 eyes with VMTS who underwent SD-OCT and reported 17% of the eyes that developed complete PVD, 17% of the eyes that formed FMH, and 65% the of eyes with persistent VMT. Codenotti et al. [13] analyzed the course of 26 eyes with VMTS for a median of 13 months and reported 23% of eyes with spontaneous resolution and 77% of eyes that underwent surgery or remained stable. When considering larger cohorts of patients, our team previously studied 183 eyes with VMTS for a minimum period of 6 months and reported resolution of the vitreomacular traction in 20% of the cases, occurring on an average at 15 months, persistent VMT in 60% of the cases, development of macular hole in 12%, and surgery for symptoms of VMTS in 8% of cases [15]. Tzu et al. [4] studied 230 eyes for a mean follow-up of 32 months and documented spontaneous release of traction in 31.7% of cases. Stalmans [14] observed a complete resolution in 22.7% of 203 eyes with VMT in a mean time of 11 months, a FTMH formation in 5.4%, and 25.6% of the eyes underwent vitrectomy due to progression.

Sonmez et al. [16], in a retrospective review of 24 cases with VMT that underwent vitrectomy, suggested that the severity of the foveal deformation may be associated with the morphological characteristics of the vitreofoveal adhesion. They suggested that a preoperative classification of the adhesion to *V*-shaped and *J*-shaped, based on the OCT, may predict the postoperative visual outcome. Since stress (*σ*) can be equated to the force (*F*) applied per cross-sectional area (*A*) perpendicular to the force (*σ* = *F*/*A*) [16], it is expected that there is greater tractional force in the eyes with a focal attachment, as the angle of the vector is more perpendicular to the retinal surface. Kozak et al. [17] analyzed retrospectively 61 eyes with VMT. They concluded that the angle of VMT is associated with increased CMT and that eyes with broader adhesion have thicker choroid when compared to those with open vitreomacular angle.

We analyzed the initial findings on OCT and their association with clinical course. We identified that the only OCT measurement that was associated with the resolution of VMT was the central macular thickness. Cases with thinner fovea at baseline had a higher risk of resolution and a lower risk of stabilization or progression to FTMH. Surprisingly, we found no association between the horizontal and vertical areas of attachment or the vertical and temporal angles of attachment and the possibility of resolution of the VMTS. That implies that a greater tractional force with a more perpendicular angle of the vector to the affected retinal surface does not affect the final anatomic configuration of the fovea. On the other hand, greater CMT, hence greater stress concentrated at the macula, is expected to cause more macular deformation and a greater possibility of persistence of the traction or progression to FTMH.

Taking into account the clinical parameters we included in our study; our analysis showed that there is a significant association between the baseline vision and either resolution of the traction, stability, or progression to a FTMH. Those with better vision had higher rates of resolution and lower rates of progression than those cases with poorer vision. This is in accordance with clinical practice where patients with poorer vision more frequently require pars plana vitrectomy for symptoms. As far as the demographics are concerned, we concluded that younger patients had a higher chance of resolution of the VMT.

Our group has previously published similar results for patients undergoing surgery for vitreomacular traction [18]. It was demonstrated that the preoperative BCVA plays a predictive role in the surgical outcome of patients with VMT undergoing pars plana vitrectomy. No other preoperative OCT characteristics demonstrated prognostic potential.

In the present study, we also found out that baseline visual acuity in VMT cases is mainly affected by the presence of COST detachment, where there is a negative correlation. This finding suggests that an elevation of the COST line, a primary change which is probably caused by the focal traction of the vitreous at the foveal center [19], is the factor which mostly impairs visual acuity in the eyes of patients with VMTS. There is also a negative correlation between the baseline VA and the average thickness of the fovea, whereas the age of the patient and the CMT have a positive, minimal effect on baseline BCVA. Finally, our analysis showed that the final visual outcome was affected only by baseline BCVA, irrespective of all the other parameters and the morphological characteristics of the traction. Our findings are in accordance with Wu et al. [20] who report a spontaneous resolution rate of 21.4%.

There are obvious limitations to our study. It is a retrospective study, which requires additional prospective data. We studied mainly patients with symptomatic VMTS who seek medical advice in contrast to patients with milder forms of VMT who do not experience any clinical symptom. Also, patients with epiretinal membrane were included in the analysis. These factors may create a reporting bias, skewing outcomes toward the more severe symptomatic cases. Visual function was assessed taking into consideration only the BCVA, which does not reflect qualitative variables of vision such as metamorphopsia or contrast sensitivity, and there is also an amount of intercorrelation between variables which could create interpretation bias. In addition, it is impossible to establish with certainty the time of disease onset. Also, the method for measurement of the angle is not proven to have high reproducibility or reliability. Nonetheless, this study demonstrates whether the angle (measured by the specific method in a pragmatic way) was found to affect the clinical course of VMT.

To our knowledge, it is the first time some of the factors are assessed for such a large case series regarding the potential prognostic factors affecting the natural course of vitreomacular traction syndrome.

## Figures and Tables

**Table 1:** Demographic and clinical characteristics.

Risk factor	Summary statistics (*n* = 150) Mean values
Age (years)	71.93
Gender	
Females	93 (62%)
Males	57 (38%)
Pre-BCVA (logMAR)	0.26
Missing	8
Post-BCVA (logMAR)	0.28
Missing	7
Follow-up (months)	20.09
Missing	3
CMT (*μ*m)	308.82
Missing	2
Volume (mm^3^)	7.5
Missing	2
Average thickness (*μ*m)	267.06
Missing	2
Horizontal area (*μ*m)	615.37
Vertical area (*μ*m)	0.92
Cost detachment	
Yes, *n* (%)	23.3
Hyaloid hyperreflectivity	
Yes, *n* (%)	18
Nasal angle (degrees)	29.38
Missing	50
Temporal angle (degrees)	30.14
Missing	50
Mean angle (*N* + *T*/2) (degrees)	32.32
ERM	
Yes, *n* (%)	14.7
Diabetes	
Yes, *n* (%)	10
Macular hole in the other eye	
Yes, *n* (%)	11.3

## Data Availability

The data used to support the findings of this study are available from the corresponding author upon request.
